# Ternary metal oxide nanocomposite for room temperature H_2_S and SO_2_ gas removal in wet conditions

**DOI:** 10.1038/s41598-022-19800-6

**Published:** 2022-09-13

**Authors:** Nishesh Kumar Gupta, Eun Ji Kim, Soyoung Baek, Jiyeol Bae, Kwang Soo Kim

**Affiliations:** 1 Department of Environmental Research, University of Science and Technology (UST), Daejeon, 34113 Korea; 2 Department of Environmental Research, Korea Institute of Civil Engineering and Building Technology (KICT), Goyang, 10223 Korea

**Keywords:** Environmental chemistry, Inorganic chemistry, Materials chemistry

## Abstract

A ternary Mn–Zn–Fe oxide nanocomposite was fabricated by a one-step coprecipitation method for the remotion of H_2_S and SO_2_ gases at room temperature. The nanocomposite has ZnO, MnO_2_, and ferrites with a surface area of 21.03 m^2^ g^−1^. The adsorbent was effective in mineralizing acidic sulfurous gases better in wet conditions. The material exhibited a maximum H_2_S and SO_2_ removal capacity of 1.31 and 0.49 mmol g^−1^, respectively, in the optimized experimental conditions. The spectroscopic analyses confirmed the formation of sulfide, sulfur, and sulfite as the mineralized products of H_2_S. Additionally, the nanocomposite could convert SO_2_ to sulfate as the sole oxidation by-product. The oxidation of these toxic gases was driven by the dissolution and dissociation of gas molecules in surface adsorbed water, followed by the redox behaviour of transition metal ions in the presence of molecular oxygen and water. Thus, the study presented a potential nanocomposite adsorbent for deep desulfurization applications.

## Introduction

Air contamination is a global issue which has been amplified by various anthropogenic activities in the last many decades. Among numerous air contaminants toxifying the air, hydrogen sulfide (H_2_S) and sulfur dioxide (SO_2_) has known to cause severe damage to human health and the environment. H_2_S is a toxic pungent-smelling gas released from decayed organic matter, oil industry, coal and natural gas-based thermal power plants, and sewage treatment facilities^1,2^. Acute exposure to H_2_S at levels of 200–500 ppm could paralyze the olfactory nerve and beyond 500 ppm could lead to sudden death^2,3^. Furthermore, H_2_S conversion to SO_2_ and its hydrolysis to form acid rain could acidify soil and water bodies, which could be disastrous to plants and marine life, respectively^4,5^. SO_2_ is a colourless toxic gas with a sharp odour, which could cause various respiratory ailments such as chronic bronchitis and infections of the respiratory tract^6^. While a low SO_2_ concentration of 1–5 ppm is enough for human discomfort, exposure above 100 ppm could be life-threatening^7^. The main sources of atmospheric SO_2_ are thermal power plants and vehicular emissions^8^. Thus, H_2_S and SO_2_ removal from point of origin should be prioritized to limit air contamination and prevent catastrophic events like smog formation and acid rain.

Chemical adsorption of these toxic gases over an adsorbent surface is one of the most simplistic and affordable methods to adsorb and mineralize H_2_S and SO_2_ gases to non-toxic by-products like sulfur and sulfates^9^. Moreover, chemisorption is highly efficient for flue gas desulfurization and natural gas purification applications, which are challenging, fundamentally and monetary-wise^1,10^. For this purpose, metal oxides have shown great potential due to the presence of weak basic sites (lattice oxygen) and basic OH^–^ groups, which could react with acidic gases like H_2_S and SO_2_ (acting as electron donors)^11,12^. The surface reactivity of metal oxides for these gases could be amplified in the presence of water molecules. Firstly, the water layer on the metal oxide surface dissociatively reacts and improves the hydroxyl density. Secondly, the surface water film dissolves the gas molecules, which lowers the energy barrier for reactive interaction with the metal oxide surface and thus favours the overall chemisorption process^13–17^. Thus, it is worth exploring the positive effect of water during the adsorption of acidic gases over metal oxides, which is the focus of this research work. Also, it is equally important to explore adsorbent materials for the remediation of low H_2_S/SO_2_ concentrates to confirm the applicability of adsorbents in deep desulfurization and gas purification applications.

In this study, we have fabricated an affordable Mn-Zn-Fe metal oxide nanocomposite by a one-step coprecipitation method for room-temperature adsorptive removal of H_2_S and SO_2_ gases in wet conditions. The gas concentration of 500 and 100 ppm for H_2_S and SO_2_ was adopted for their industrial application and suitability in capturing these pollutants in the toxicity range for humans. The oxide showed better adsorption performance in wet conditions with complete mineralization to non-toxic by-products. Besides studying the factors affecting the adsorption process, the adsorption mechanism was studied in detail using various microscopic and spectroscopic techniques. The study confirmed that the oxide nanocomposite has the potential to eliminate and mineralize low concentrations of gaseous H_2_S and SO_2_ in dry–wet conditions.

## Methods

### Chemicals

Manganese(II) nitrate tetrahydrate (Mn(NO_3_)_2_·4H_2_O), zinc(II) nitrate hexahydrate (Zn(NO_3_)_2_·6H_2_O), iron(III) nitrate nonahydrate (Fe(NO_3_)_3_·9H_2_O), and 2.0 mol L^−1^ NaOH solution were procured from Samchum Pure Chemicals, Korea. H_2_S gas (0.05 Vol.%) and SO_2_ gas (0.01 Vol.%) balanced with pure N_2_ gas were procured from Union gas, Korea. All solutions were prepared in double-distilled water.

### Synthesis of nanocomposite

In 50 mL of deionized water, 3.76 g of Mn(NO_3_)_2_·4H_2_O, 4.45 g of Zn(NO_3_)_2_·6H_2_O, and 4.04 g of Fe(NO_3_)_3_·9H_2_O were dissolved to give a final Mn^2+^:Zn^2+^:Fe^3+^ ratio of 3:3:2. A higher ratio of divalent to trivalent cations was adopted for the formation of MnO_2_ and ZnO in the nanocomposite (for a higher acidic gas adsorption capacity). Under vigorous stirring, 2.0 mol L^−1^ NaOH solution was added dropwise until the solution pH reached 12.5. This pH was sufficient for the formation of ternary oxide nanocomposites as reported earlier^18^. After stirring for 2 h, the precipitate was phase separated and dried at 393 K overnight in a hot air oven. The use of an excess of water for washing was avoided to reduce the overall impact on the environment during the material fabrication process.

### Analytical instruments

The morphology in the surface and transmission mode was probed over field emission scanning electron microscopy (FE-SEM, Hitachi S-4300, Hitachi, Japan) and field emission TEM (FE-TEM, JEM-2010F, JEOL Ltd., Japan), respectively. SEM analysis was done on finely grounded dried samples after coating them with a gold-platinum alloy by ion-sputtering (E-1048 Hitachi ion sputter). The elemental analysis was done using energy-dispersive X-ray spectroscopy (EDAX, X-Maxn 80 T, Oxford Instruments, United Kingdom). The specific surface area and porosity were determined by analysing the standard N_2_ adsorption–desorption isotherm at 77 K using a Gemini 2360 series (Micromeritics, Norcross, United States) instrument after degassing at 423 K for 6 h with a mass of 0.324 g. The powder X-ray diffraction (PXRD) patterns were obtained at room temperature (2θ = 5–50°) on an Ultima IV (Rigaku, Japan) X-ray diffractometer with Cu Kα radiation (λ = 1.5406 Å) and a Ni filter. Fourier-transform infrared (FTIR) spectra of samples were recorded using KBr pellets over a Cary670 FTIR spectrometer (Agilent Technologies, United States). For X-ray photoelectron spectroscopy (XPS) analysis, a K-alpha XPS instrument (Thermo Fisher Scientific, United Kingdom) was used with a monochromatic Al K_α_ X-ray source. The pressure was fixed to 4.8 × 10^−9^ mbar. Spectra were charge corrected to the main line of the C 1 s (aromatic carbon) set to 284.7 eV. Spectra were analysed using CasaXPS software (version 2.3.14).

### Breakthrough protocol

The gas adsorption experiments were performed by taking 0.5 g of the adsorbent in a Pyrex tube (height: 50 cm and diameter: 1 cm) at 298 K. The sample was fixed between the glass wool and supported on silica beads^19^. The H_2_S gas (0.05 Vol.%) or SO_2_ gas (0.01 Vol.%) was passed through it at a fixed flow rate. The outgoing gas was analyzed using an H_2_S gas analyzer (GSR-310, Sensoronic, Korea) or an SO_2_ gas analyzer (GASTIGER 6000, Wandi, Korea). The analyzer recorded the effluent gas concentration every minute in real-time until the breakthrough points of 20% (100 ppm for H_2_S and 20 ppm for SO_2_) were reached and after that the experiment was completed. The wet samples were prepared by passing water vapours (80% relative humidity) directly through the adsorbent bed before passing the gas through it. The gas adsorption capacity was measured using the following equation:

1 $$ q = \frac{{C_{0} Q}}{m}\mathop \int \limits_{0}^{{t_{b} }} \left( {1 - \frac{{C_{t} }}{{C_{0} }}} \right)dt $$

where *C*_0_-initial concentration (mg L^−1^), *C*-concentration at time ‘*t*’ (mg L^−1^), *Q*-flowrate (L min^−1^), *m*-the mass of adsorbent (g), and *t*_b_-breakthrough time (s).

## Results and discussion

**Figure 1:**
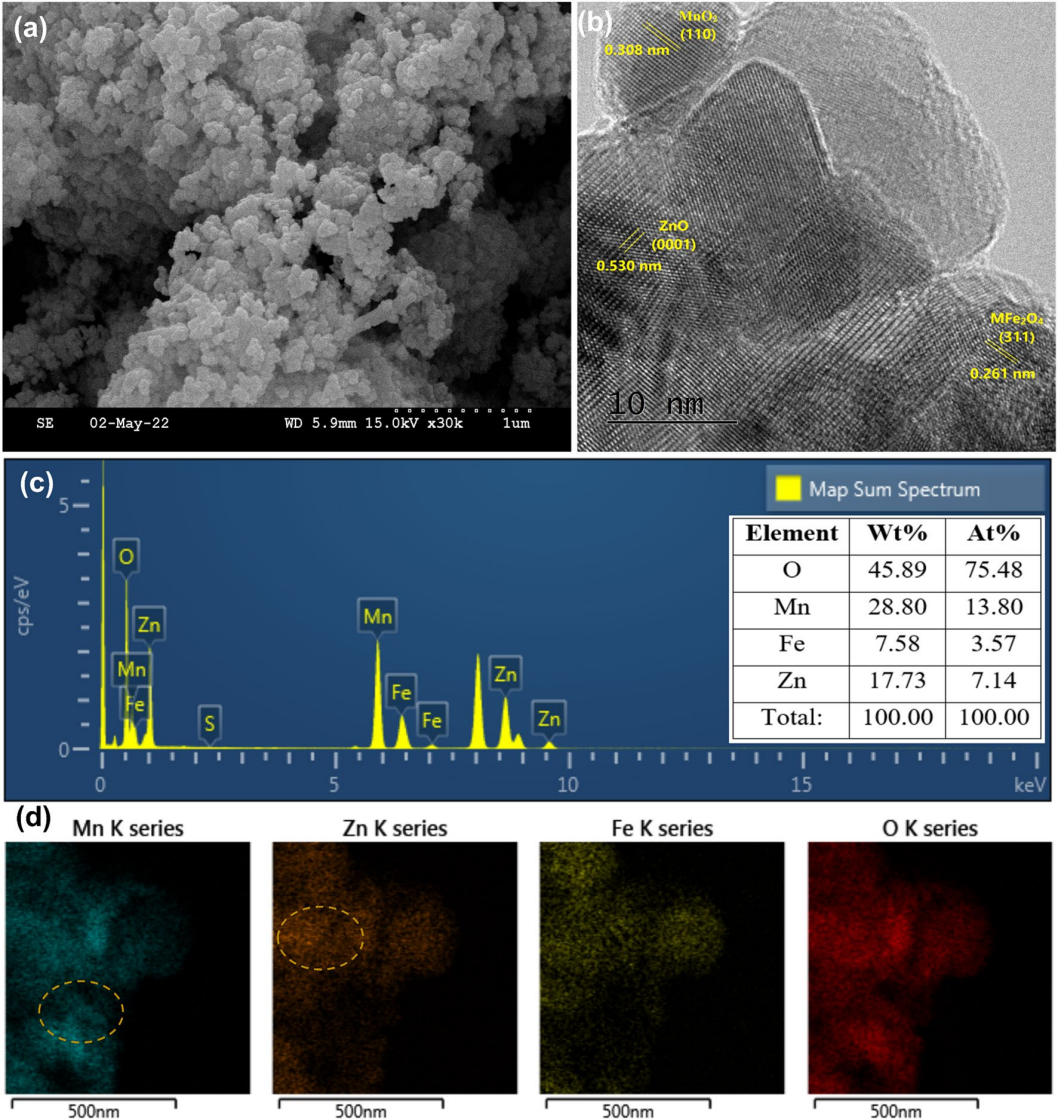
(**a**) SEM micrograph; (**b**) high-resolution TEM micrograph; (**c**) elemental distribution analysis; (**d**) 2D elemental mapping of MZFO.

The SEM micrograph of oxide nanocomposite showed irregularly shaped nano-globules, which were uniformly distributed in the entire region (Fig. 1a). The controlled release of base (precipitation agent) assisted in regulating the nucleation and particle growth kinetics, which prevented the aggregation of metal oxides in the ternary nanocomposite. A more detailed investigation of morphology was conducted over high-resolution TEM, which confirmed that the nano-globules were constructed of polyhedral nanoparticles (Fig. 1b). The crystallite planes of nanoparticles were assigned by measuring the fringe width and correlating with the interplanar spacing (*d*) values from the XRD pattern. The fringe width of 0.308, 0.530, and 0.261 nm were assigned to the MnO_2_ (110)^20^, ZnO (0002)^21^, and MFe_2_O_4_ (311)^22^, respectively. The EDAX elemental analysis confirmed peaks for Mn, Zn, Fe, and O at respective energies having the atomic contribution of 13.80, 7.14, 3.57, and 75.48%, respectively (Fig. 1c). The 2D elemental mapping showed an abundant density of Mn and Zn with low-density regions in the ‘Fe’ map (respective high-density regions marked in ‘Mn’ and ‘Zn’ maps). The possible reason for such a distribution could be the formation of pure, binary, and ternary metal oxides in the nanocomposite (Fig. 1d).

**Figure 2:**
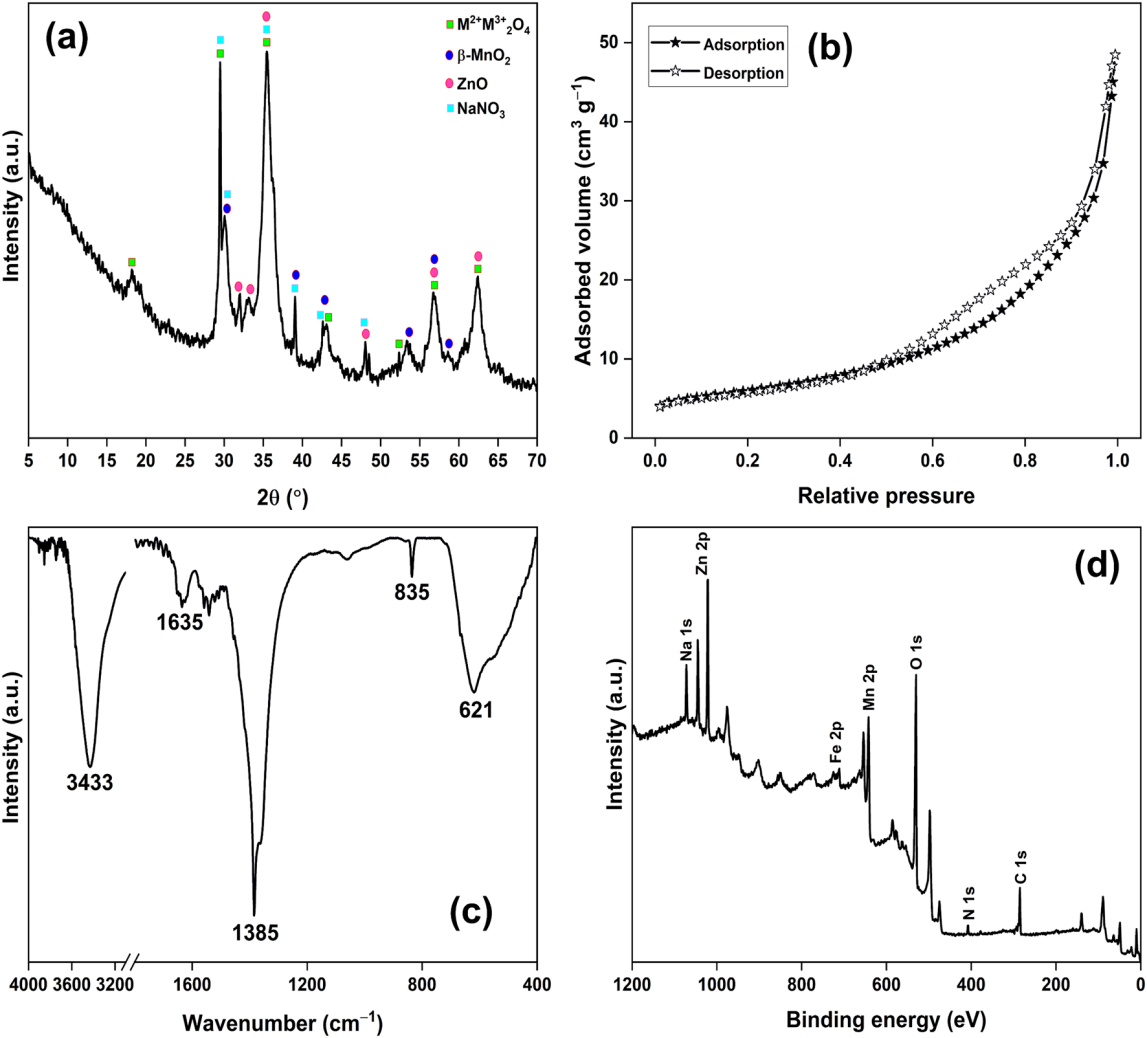
(**a**) PXRD pattern; (**b**) N_2_ adsorption–desorption isotherm; (**c**) FTIR spectrum; (**d**) XPS survey of MZFO.

The PXRD pattern of MFZO nanocomposite has diffraction peaks for β-MnO_2_ (purple circle)^23^, ZnO (pink circle)^24^, ferrites (green square)^25^, and NaNO_3_ (blue square)^26^, which confirmed a poly-oxide nature of the composite (Fig. 2a). A similar report is available for the fabrication of Cu–Zn-Mn ternary oxide nanocomposite, where CuO, ZnO, and MnO_2_ nanoparticles were confirmed^18^. The absence of a washing step during the MFZO fabrication was responsible for the presence of NaNO_3_ in the sample. The N_2_ adsorption–desorption isotherm of MFZO nanocomposite exhibited a Type III behaviour, generally expected in macro-porous materials (Fig. 2b)^23^. The nanocomposite possessed a BET surface area of 21.03 m^2^ g^−1^ and a pore volume of 0.07 cm^3^ g^−1^. These values are higher than other nanocomposites like Mn_2_O_3_/Fe_2_O_3_ (6.18 m^2^ g^−1^, 0.12 cm^3^ g^−1^)^27^, CeO_2_/Mn_2_O_3_/Fe_2_O_3_ (15.64 m^2^ g^−1^, 0.09 cm^3^ g^−1^)^28^, and Fe_2_O_3_/Na_2_SO_4_ (2.89 m^2^ g^−1^, 0.01 cm^3^ g^−1^)^29^ used for the same applications. The spectrum has a broad band centred at 621 cm^−1^ for the metal–oxygen stretching vibrations^30,31^. The bands at 835 and 1385 cm^−1^ were attributed to the asymmetric stretching vibration (*ν*_3_-NO_3_^−^) and out-of-plane bending vibration (*ν*_2_-NO_3_^−^), respectively, of nitrate^32^. The bands at 3433 and 1635 cm^−1^ were assigned to the stretching and bending vibration modes of adsorbed water molecules, respectively (Fig. 2c)^33^. The XPS survey of MFZO confirmed peaks for Na 1 s, Zn 2p, Fe 2p, Mn 2p, O 1 s, and N 1 s at their respective binding energy. The Na 1 s and N 1 s peaks were associated with the presence of NaNO_3_ (Fig. 2d).

**Figure 3:**
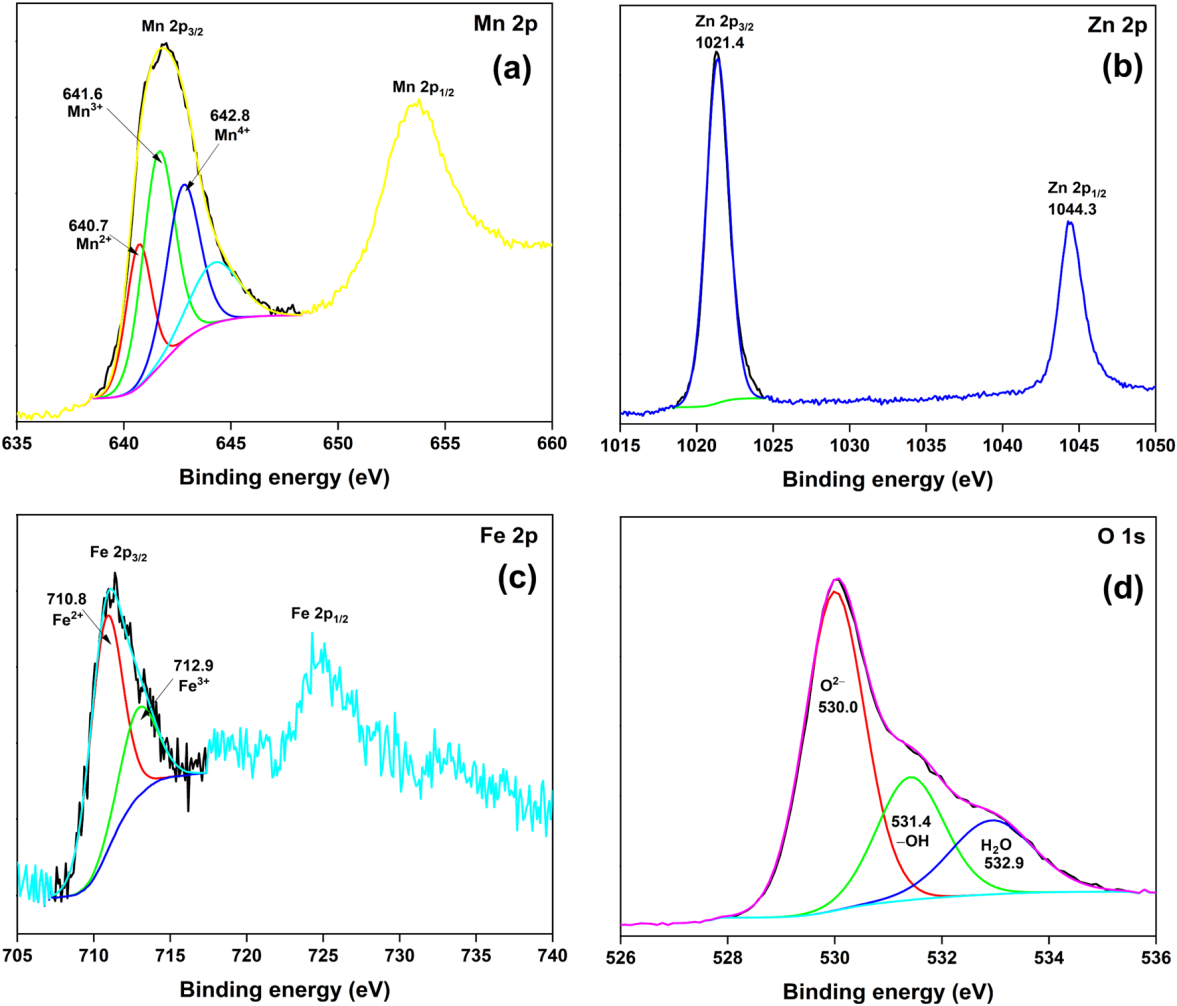
High-resolution XPS (**a**) Mn 2p; (**b**) Zn 2p; (**c**) Fe 2p; (**d**) O 1 s spectra of MFZO.

The HRXPS Mn 2p_3/2_ signal of MFZO deconvoluted into three contributions at 640.7, 641.6, and 642.8 eV for Mn^2+^ (23.2%), Mn^3+^ (43.9%), and Mn^4+^ (32.9%) oxidation states of Mn ions, respectively^34^. The analyses showed that multivalent Mn ions were related to the formation of MnO_2_ and Mn-based ferrites (Fig. 3a, Table S1). The HRXPS Zn 2p spectrum has two peaks at 1021.4 and 1044.3 eV for 2p_3/2_ and 2p_1/2_ signals of Zn^2+^ ions, respectively (Fig. 3b, Table S2)^35^. In the HRXPS Fe 2p spectrum, the 2p_3/2_ signal was deconvoluted into two contributions at 710.8 and 712.9 eV for Fe^2+^ (70.4%) and Fe^3+^ ions (29.6%), respectively (Fig. 3c, Table S3)^36^. These two contributions were due to the formation of ferrites. The HRXPS O 1 s spectrum has three deconvoluted peaks at 530.0, 531.4, and 532.9 eV for lattice oxygen (56.9%), surface hydroxyl groups/nitrate ions (24.6%), and water molecules (18.4%), respectively (Fig. 3d, Table S4)^37^.

**Figure 4:**
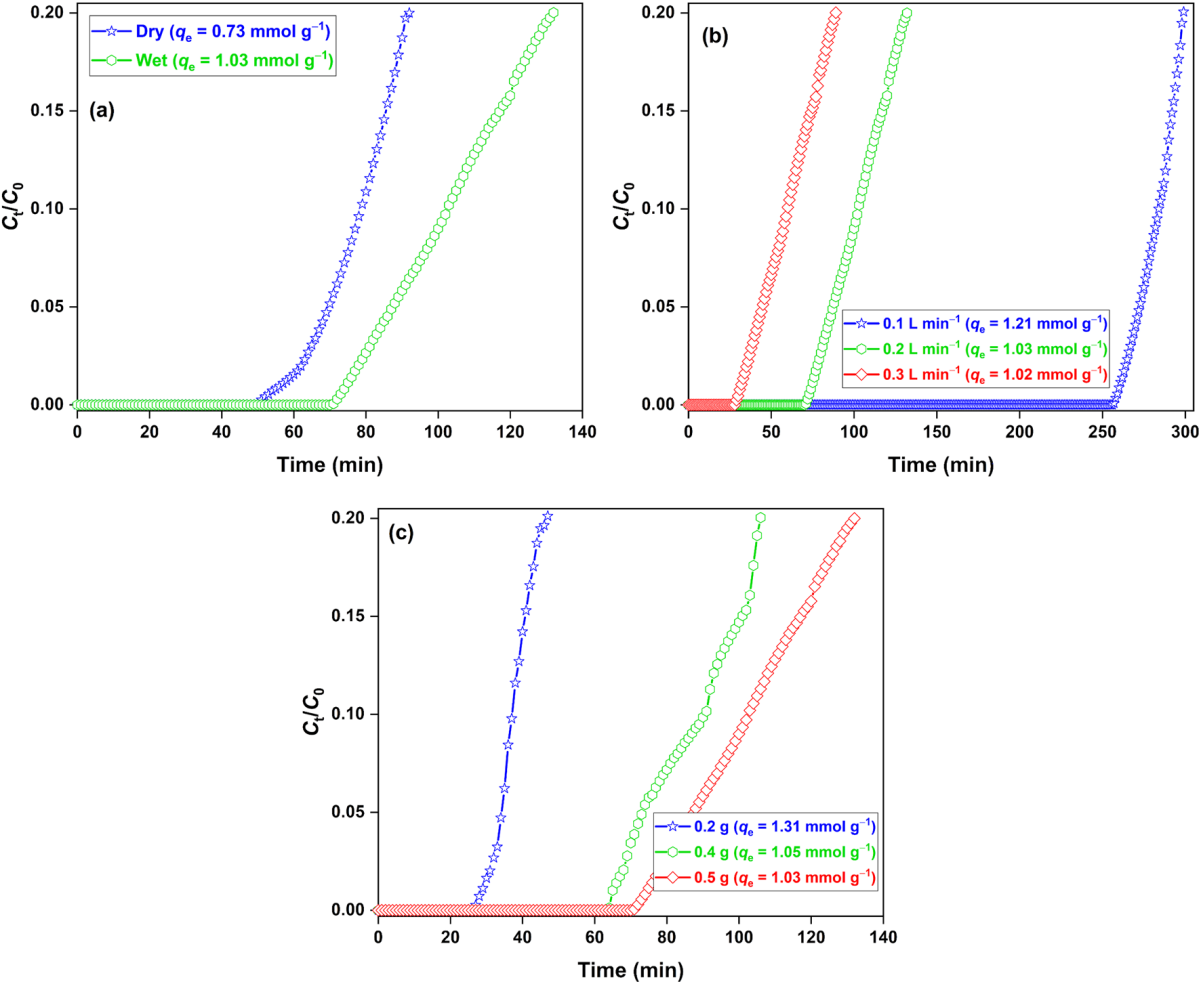
H_2_S breakthrough curves for (**a**) dry/wet adsorbents; (**b**) wet adsorbent at different flowrate; (**c**) adsorbent loading. Conditions: [Adsorbent] = 0.5 g, flowrate = 0.2 L min^−1^ (changed otherwise).

The synthesized nanocomposite was tested for H_2_S removal in breakthrough columns both in dry and wet conditions (Fig. 4a). The adsorption capacity of 0.73 mmol g^−1^ was achieved in the dry condition. However, in the wet condition, the capacity increased to 1.03 mmol g^−1^, showing the positive role of water in the adsorption process mediated by the dissolution and dissociation of H_2_S molecules in the water film over the oxide surface. The effect of parameters, *i.e.*, gas flow rate (Fig. 4b) and adsorbent loading (Fig. 4c) on the adsorption capacity was studied in the wet conditions. The adsorption capacity decreased with the increasing flow rate, where the highest capacity of 1.21 mmol g^−1^ was achieved with a flow rate of 0.1 L min^−1^. Increasing flow rate disfavoured the adsorbate-adsorbent interaction due to a lowering in the gas retention time, which negatively impacted the capacity^38^. The adsorption capacity decreased with the increasing adsorbent loading and the maximum capacity of 1.31 mmol g^−1^ was achieved for 0.2 g of adsorbent and 0.2 L min^−1^ of flowrate. This behaviour could be associated with the presence of an unutilized mass of the oxide likely due to the cluttering of wet adsorbent particles in the adsorbent bed, which reduces the effective surface area for the reaction to occur^31^. However, no breakthrough experiment was conducted below 0.2 g. Since the material has a high density, loading adsorbent below 0.2 g led to a narrow bed length (since the tube diameter was 6 mm), which had a poor adsorbate-adsorbent interaction. The maximum adsorption capacity of 1.31 mmol g^−1^ achieved for the synthesized nanocomposite is similar to or higher than those reported for commercial ZnO (1.16 mmol g^−1^)^39^, CeO_2_-Mn_2_O_3_-Fe_2_O_3_ (0.83 mmol g^−1^)^28^, Mn_2_O_3_-Fe_2_O_3_ (0.35 mmol g^−1^)^27^, α-Fe_2_O_3_-Na_2_SO_4_ (1.06 mmol g^−1^)^29^, and ZnFe_2_O_4_ (0.05 mmol g^−1^)^40^ in similar experimental conditions.

**Figure 5:**
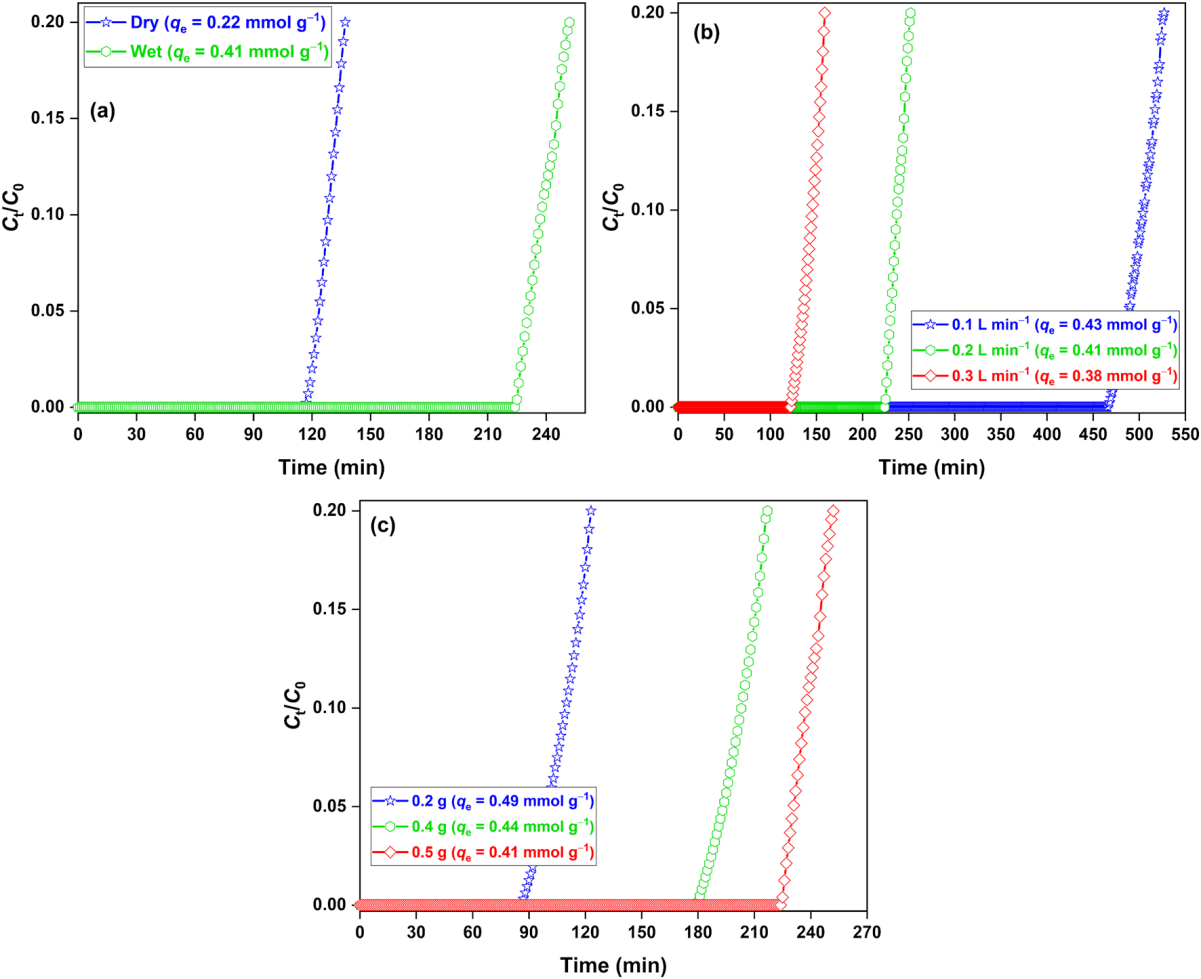
SO_2_ breakthrough curves for (**a**) dry/wet adsorbent; (**b**) wet adsorbent at different flowrate; (**c**) wet adsorbent at different adsorbent loading. Conditions: [Adsorbent] = 0.5 g, flowrate = 0.2 L min^−1^ (changed otherwise).

The nanocomposite was also studied for SO_2_ adsorption in dry and wet conditions (Fig. 5a). The adsorption capacity of 0.22 mmol g^−1^ in the dry condition nearly doubled to 0.41 mmol g^−1^ in the wet condition. Such behaviour has been reported for oxidative SO_2_ adsorption over MnO_2_^16^. SO_2_ adsorption in the presence of water molecules significantly accelerated the sulfate formation reaction, which favoured the overall adsorption process. The increasing gas flow rate negatively affected the adsorption process due to poor adsorbate-adsorbent interaction (Fig. 5b). The SO_2_ adsorption capacity of the composite significantly improved with the decreasing adsorbent loading, where the maximum adsorption capacity of 0.49 mmol g^−1^ was confirmed with 0.2 g of adsorbent and 0.2 L min^−1^ of flowrate. Here also, the negative role of increasing bed loading was according to the effect witnessed for H_2_S gas adsorption (Fig. 5c). Thus, in the optimized experimental conditions, the adsorbent could remove 0.49 mmol g^−1^ of SO_2_ gas. This value is highly significant and comparable to the reported values for ZnO (0.28 mmol g^−1^)^41^, MnO_2_ (0.48–1.23 mmol g^−1^)^42^, and NaM_x_O_y_ (0.73 mmol g^−1^)^7^ in similar experimental conditions. The nanocomposite possessed a higher H_2_S adsorption capacity compared to the SO_2_ gas. The superior H_2_S adsorption was related to an easy dissociation of H_2_S molecules due to the much lower energy barrier and higher adsorption energy compared to SO_2_, which has been previously demonstrated for Zn–MoSe_2_ structure through computational calculations^43^.

**Figure 6:**
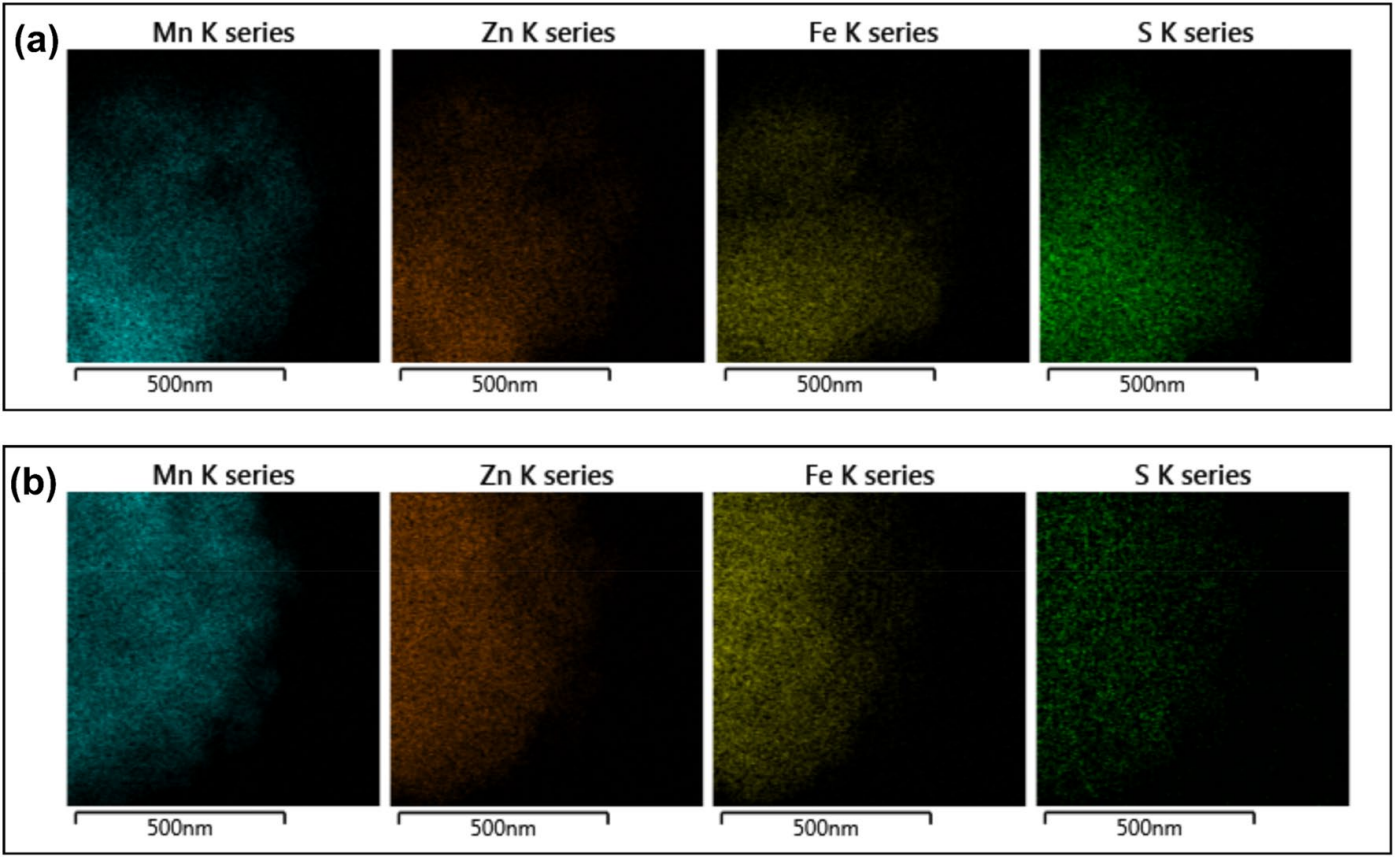
2D elemental mapping of wet MFZO after (**a**) H_2_S; (**b**) SO_2_ adsorption.

The SEM and TEM micrographs post-H_2_S and SO_2_ adsorption showed no significant variation in the surface morphology (Figs. S1, S2) except for the transformation of a chunk of nanoparticles to cluttered nanorods. It could be due to the combined effect of moisture and gas acidity as the SEM micrographs of dry samples showed no such change in the surface morphology. The EDAX analysis of gas-exposed samples confirmed a new peak at ~ 2.3 keV for sulfur. The intensity of the S peak in the H_2_S-adsorbed sample is much higher than that of the SO_2_, which agreed with the experimental results (Fig. S3). The 2D elemental mapping of the H_2_S and SO_2_-adsorbed samples confirmed a high density of sulfur atoms over the oxide surface, which was uniformly distributed over the nanocomposite (Fig. 6).

**Figure 7:**
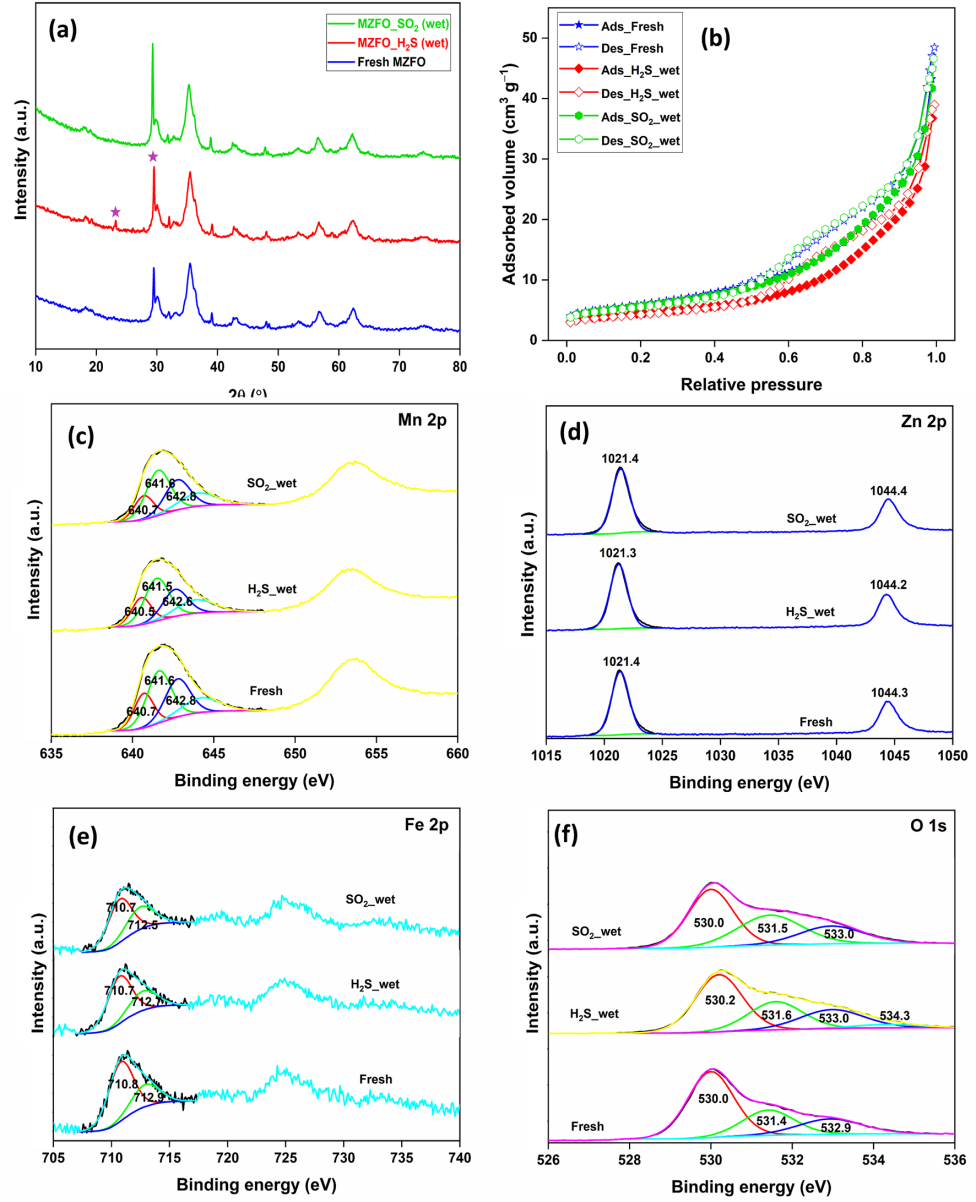
(**a**) PXRD patterns; (**b**) N_2_ adsorption–desorption isotherms; High-resolution XPS (**c**) Mn 2p; (**d**) Zn 2p; (**e**) Fe 2p; (**f**) O 1 s spectra of wet MZFO after H_2_S and SO_2_ adsorption.

The PXRD patterns of gas-exposed samples showed insignificant changes in the diffraction peaks, except for new peaks in the H_2_S-adsorbed sample (marked as purple stars). These two peaks were assigned to the presence of ZnSO_3_^44^. The absence of additional new peaks in these samples could be related to the formation of oxidized sulfur species on the surface (Fig. 7a). The N_2_ adsorption–desorption isotherms are shown in Fig. 7b. For the H_2_S-adsorbed sample, the surface area and pore volume decreased by 26 and 17%, respectively (Table 1). However, for the SO_2_-exposed sample, a minimal drop in these values was observed. The drop in the surface area and porosity was linked to the deposition of oxidized sulfur species, which may have clogged the pores^29^. This clogging was expected more in the H_2_S-adsorbed sample due to a higher gas volume adsorption and subsequent mineralization onto the surface.

**Table 1:** Surface area and pore characteristics of wet MFZO before and after gas adsorption.

Sample	Surface area (m^2^ g^−1^)	Pore volume (cm^3^ g^−1^)	Average pore diameter (nm)
Fresh MFZO	21.03	0.070	13.4
MFZO_H_2_S	15.56	0.058	14.8
MFZO_SO_2_	20.16	0.068	13.4

More detailed information on the adsorption mechanism was deduced for the XPS analysis of MFZO nanocomposite after the gas adsorption process. In the HRXPS Mn 2p spectrum of the H_2_S-adsorbed sample, all three contributions for Mn^2+^, Mn^3+^, and Mn^4+^ are present at a slightly lower binding energy with variation in the proportion of these oxidation species. The redshift in the binding energy could be associated with the partial sulfidation of the Mn oxides^31^. Moreover, the variation in the oxidation state proportions could be linked to the involvement of Mn^2+^/Mn^3+^/Mn^4+^ redox cycles during the chemisorption process^17^. However, for SO_2_-adsorbed samples, the only proportion of oxidation states varied with an insignificant shift in the position, which was related to the Mn redox behaviour responsible for the oxidation of SO_2_ (Fig. 7c)^17,45^. The HRXPS Zn 2p spectrum of H_2_S-adsorbed showed a minimal redshift in the peak position probably due to the formation of ZnSO_3_ species. However, no such shift was witnessed for the SO_2_-adsorbed sample, which further suggested the delocalized nature of the chemisorption process (Fig. 7d). In the HRXPS Fe 2p spectrum of H_2_S-adsorbed MFZO, the peak position shifted slightly but with a minimal change in the proportion of Fe^2+^ and Fe^3+^ species. DFT calculations have predicted that H_2_S dissociatively reacts better on the FeO (Fe^2+^ sites) than Fe_2_O_3_ (Fe^3+^ sites)^46^. Even in our previously reported work on the adsorption of H_2_S over Mn_2_O_3_/Fe_2_O_3_, bulk Fe_2_O_3_ phase did not take part in the oxidation process^27^. The slight variation in the peak position could be linked to the involvement of Fe^2+^ sites in the H_2_S adsorption process. For the SO_2_-adsorbed sample, the Fe^2+^ and Fe^3+^ peak positions red-shifted by 0.1 and 0.4 eV, respectively, with a significant drop in the Fe^2+^ proportion (70.4–62.3%). It has been proven that the SO_2_ molecules are much more reactive to the Fe^2+^ sites than the Fe^3+^ sites. Thus, a drop in the Fe^2+^ contribution suggested that the divalent Fe sites catered the oxidation of SO_2_ molecules (Fig. 7e)^47^. In the HRXPS O 1 s spectrum of H_2_S-adsorbed sample, the metal–oxygen bond contribution decreased, whereas the contribution at 531.6 eV for −OH/O–N increased due to the formation of metal-sulfide and sulfite (SO_3_^−^) species, respectively. For the SO_2_-adsorbed sample, the 531.5 eV peak improved even further due to the consumption of lattice oxygen and the formation of sulfate species (Fig. 7f).

**Figure 8:**
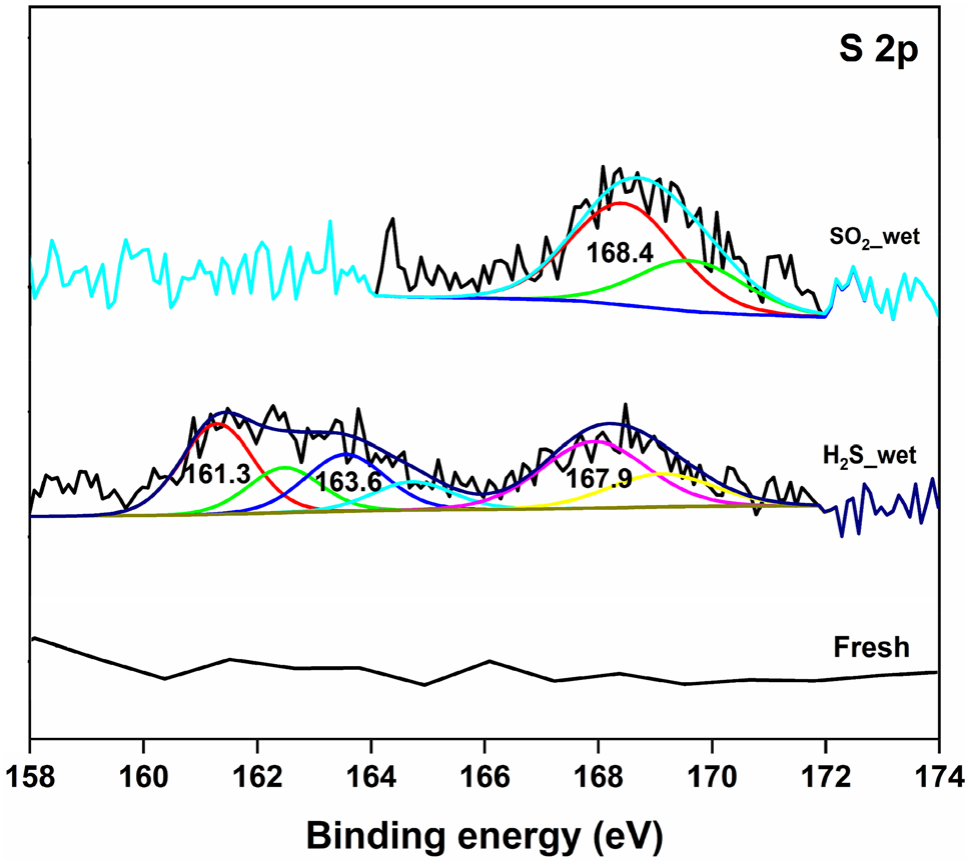
High-resolution XPS S 2p spectra of MFZO before and after H_2_S and SO_2_ adsorption.

The HRXPS S 2p spectrum of H_2_S-adsorbed MFZO was deconvoluted into three sets of doublets with their 2p_3/2_ peaks observed at 161.3, 163.6, and 167.9 eV for sulfide (36.1%), elemental sulfur (25.1%), and sulfite (38.8%)^48^. While the formation of metal-bound sulfide is initiated by the dissociated adsorption of H_2_S (into H^+^ and HS^**−**^) in the presence of water molecules. The formation of elemental sulfur and sulfide is mediated by the redox behaviour of transition metal ions, surface-adsorbed molecular oxygen, and water molecules^31^. Wang et al.have reported the dissociation of H_2_S over In_2_O_3_ thin film in the presence of moisture, where the reactive dissociation of H_2_S molecules with adsorbed water produced HS^−^ and H^+^ species. The formed HS^−^ and H^+^ ions reacted with the surface-chemisorbed oxygen species to yield sulfide and sulfite species^49^. The HRXPS S 2p spectrum of SO_2_-adsorbed MFZO has a set of doublets with a 2p_3/2_ peak at 168.4 eV, which was assigned to the sulfate species (Fig. 8, Table S5)^48^. The adsorption of SO_2_ over the oxide surface is generally driven by the reactive interaction of SO_2_ molecules with the lattice oxygen or surface hydroxyl groups to form sulfite/bisulfite, which further oxidized to sulfate via redox behaviour of transition metal oxide and gaseous oxygen molecules^7,17^. Moreover, just like H_2_S dissolution in the surface water, SO_2_ could be readily adsorbed and hydrolysed by surface water molecules, which makes the oxidation of SO_2_ molecules, energetically favourable^17^.

## Conclusion

In conclusion, we have fabricated an Mn-Zn-Fe metal oxide nanocomposite via a one-step coprecipitation reaction. The fabricated nanocomposite has MnO_2_, ZnO, and ferrites with a surface area and pore volume of 21.03 m^2^ g^−1^ and 0.07 cm^3^ g^−1^, respectively. The nanocomposite was tested for room-temperature adsorptive removal of H_2_S and SO_2_ in dry and wet conditions. The oxide exhibited better gas adsorption capacity in wet conditions owing to the dissolution and dissociation of gaseous molecules in the surface water film. The adsorbent showed a better adsorption capacity at a lower flow rate and adsorbent loading. In the optimized conditions, a maximum of 1.31 and 0.49 mmol g^−1^ of H_2_S and SO_2_ was removed by the nanocomposite, respectively. The in-depth spectroscopic analysis confirmed the mineralization of H_2_S gas into sulfide, sulfur, and sulfite, which was mediated by the Fe and Mn redox cycles in the presence of adsorbed water and molecular oxygen. Though Zn ions did not participate in the oxidation process, Zn^2+^ probably interacted with the sulfides and sulfites. The SO_2_ mineralization was associated with the formation of sulfates, driven by the redox behaviour of Fe and Mn in an oxidative environment. Thus, we have presented a novel adsorbent material for the successful mineralization of toxic sulfurous gases, which could be suitable for deep desulfurization applications.

## Supplementary Information

Supplementary Information.

## Data Availability

Data is available from the corresponding author after reasonable request.
