# Supplementary material for: Ternary metal oxide nanocomposite for room temperature H2S and SO2 gas removal in wet conditions
Source: Sci Rep. 2022 Sep 13;12:15387. doi: 10.1038/s41598-022-19800-6 (PMC9470665; doi:10.1038/s41598-022-19800-6)
Supplement: Supplementary file 1 — Supplementary Information. [file 41598_2022_19800_MOESM1_ESM.docx]

Supplementary file

Ternary Metal Oxide Nanocomposite for Room Temperature H_2_S and SO_2_ Gas Removal in Wet Conditions

Nishesh Kumar Gupta^a,b^, Eun Ji Kim^a,b^, Soyoung Baek^b^, Jiyeol Bae^a,b*^, Kwang Soo Kim^a,b**^


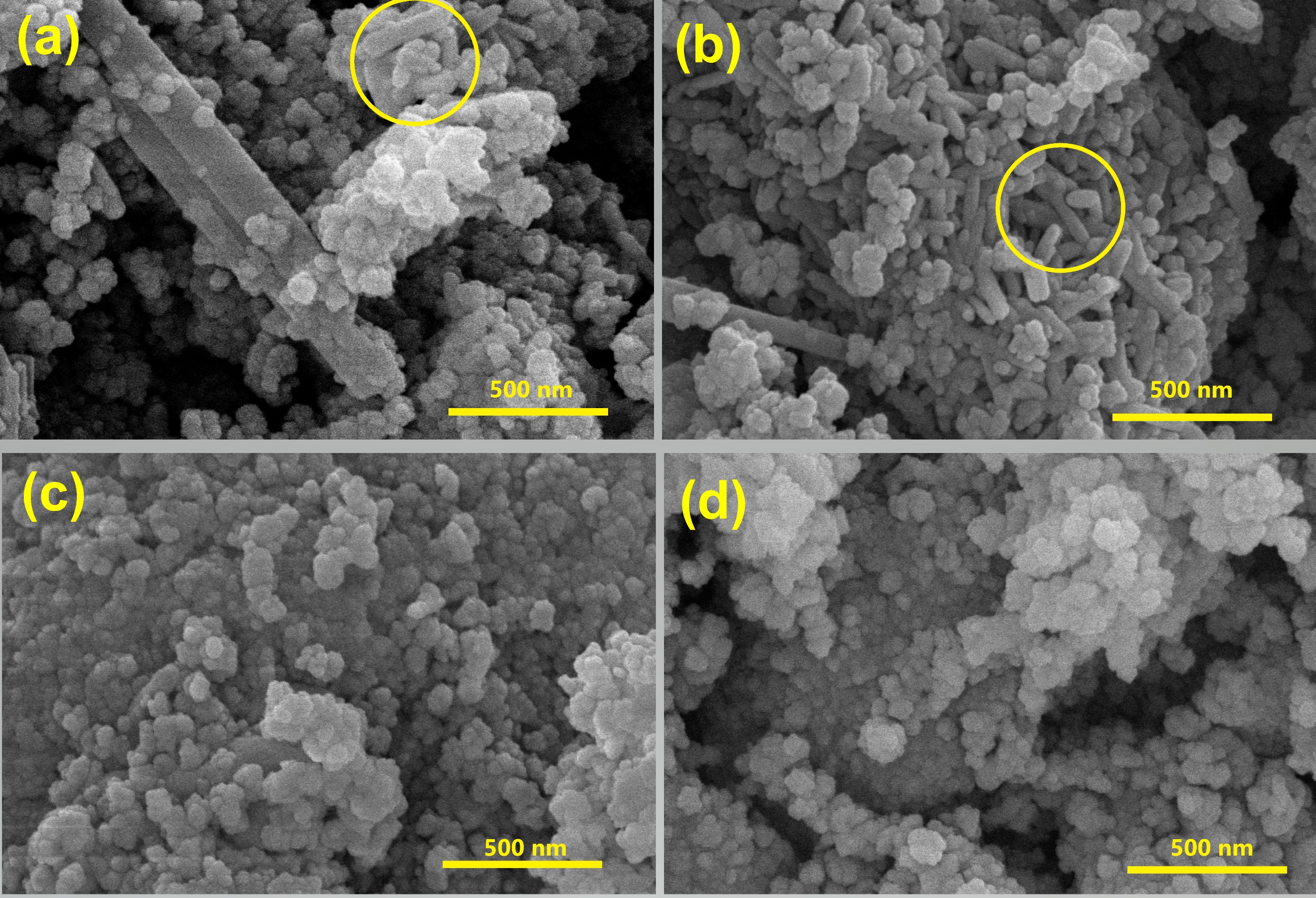


**Figure S1.** SEM micrographs of wet MZFO after (**a**) H_2_S; (**b**) SO_2_ adsorption and dry MZFO after (**c**) H_2_S; (**d**) SO_2_ adsorption.


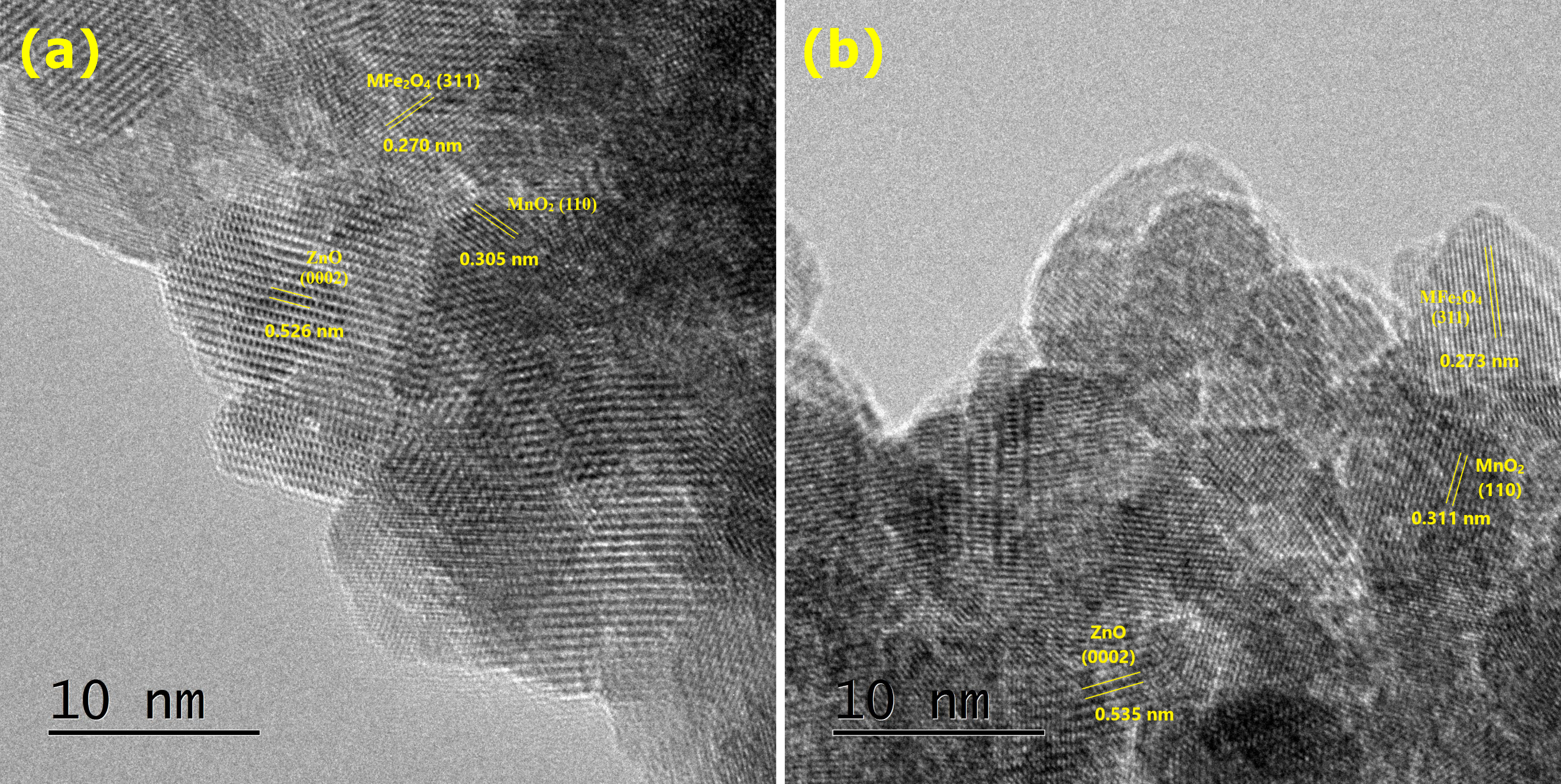


**Figure S2.** TEM micrographs of wet MZFO after (**a**) H_2_S adsorption; (**b**) SO_2_ adsorption.


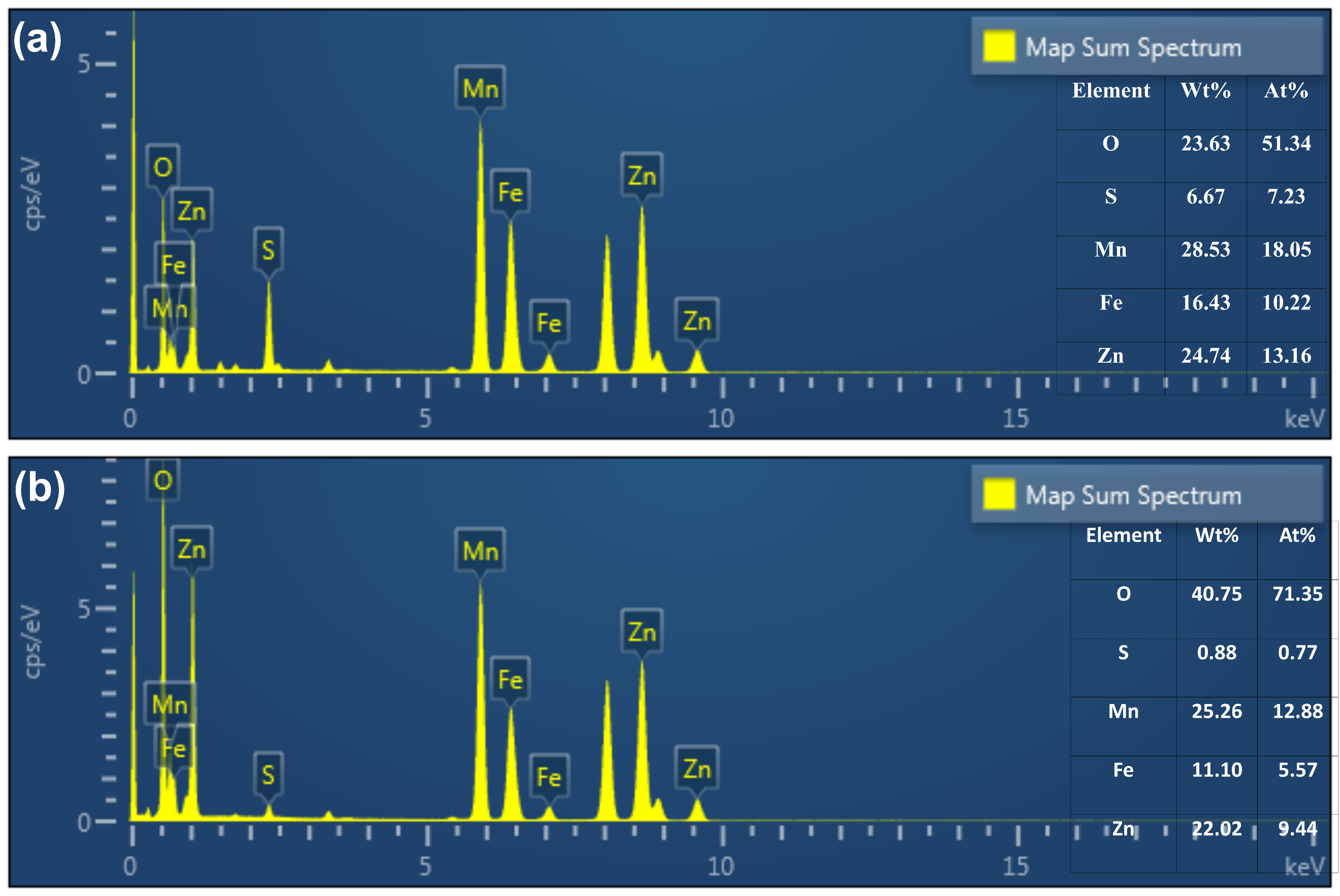


**Figure S3.** Elemental analyses of wet MZFO after (**a**) H_2_S adsorption; (**b**) SO_2_ adsorption.

**Table S1.** XPS Mn 2p assignment of MZFO before and after gas adsorption.

| **Samples** | **Assignment** | **E_B_ (eV)** | **FWHM (eV)** | **At. %** |
| --- | --- | --- | --- | --- |
| **Fresh** | **Mn2p_3/2_** _Mn2+_ | 640.7 | 1.4 | 23.3 |
|  | **Mn2p_3/2_** _Mn3+_ | 641.6 | 1.7 | 43.9 |
|  | **Mn2p_3/2_** _Mn4+_ | 642.8 | 1.8 | 32.9 |
|  | **Mn2p_3/2_** _Satellite_ | 644.2 | 2.7 | - |
| **H_2_S** | **Mn2p_3/2_** _Mn2+_ | 640.5 | 1.4 | 23.7 |
|  | **Mn2p_3/2_** _Mn3+_ | 641.5 | 1.7 | 46.5 |
|  | **Mn2p_3/2_** _Mn4+_ | 642.6 | 1.8 | 29.8 |
|  | **Mn2p_3/2_** _Satellite_ | 643.9 | 2.7 | - |
| **SO_2_** | **Mn2p_3/2_** _Mn2+_ | 640.7 | 1.4 | 20.0 |
|  | **Mn2p_3/2_** _Mn3+_ | 641.6 | 1.7 | 47.5 |
|  | **Mn2p_3/2_** _Mn4+_ | 642.8 | 1.8 | 32.5 |
|  | **Mn2p_3/2_** _Satellite_ | 644.0 | 2.7 | - |

**Table S2.** XPS Zn 2p assignment of MZFO before and after gas adsorption.

| **Samples** | **Assignment** | **E_B_ (eV)** | **FWHM (eV)** | **At. %** |
| --- | --- | --- | --- | --- |
| **MZFO** | **Zn 2p_3/2_** | 1021.4 | 1.8 | 100 |
| **MZFO_H_2_S** | **Zn 2p_3/2_** | 1021.3 | 1.8 | 100 |
| **MZFO_SO_2_** | **Zn 2p_3/2_** | 1021.4 | 1.8 | 100 |

**Table S3.** XPS Fe 2p assignment of MZFO before and after gas adsorption.

| **Samples** | **Assignment** | **E_B_ (eV)** | **FWHM (eV)** | **At. %** |
| --- | --- | --- | --- | --- |
| **MZFO** | **Fe2p_3/2_** _Fe(II)_ | 710.8 | 2.5 | 70.4 |
|  | **Fe2p_3/2_** _Fe(III)_ | 712.9 | 2.8 | 29.6 |
| **MZFO_ H_2_S** | **Fe2p_3/2_** _Fe(II)_ | 710.7 | 2.4 | 68.2 |
|  | **Fe2p_3/2_** _Fe(III)_ | 712.7 | 2.8 | 31.8 |
| **MZFO_SO_2_** | **Fe2p_3/2_** _Fe(II)_ | 710.7 | 2.4 | 62.3 |
|  | **Fe2p_3/2_** _Fe(III)_ | 712.5 | 2.8 | 37.7 |

**Table S4.** XPS O 1s assignment of MZFO before and after gas adsorption.

| **Samples** | **Assignment** | **E_B_ (eV)** | **FWHM (eV)** | **At. %** |
| --- | --- | --- | --- | --- |
| **MZFO** | **O1s** _O-Fe, O-Mn, O-Zn_ | 530.0 | 1.3 | 56.9 |
|  | **O1s** _-OH_ | 531.4 | 1.5 | 24.6 |
|  | **O1s** _-OH_ | 532.9 | 1.8 | 18.4 |
| **MZFO_ H_2_S** | **O1s** _O-Fe, O-Mn, O-Zn_ | 530.2 | 1.4 | 47.6 |
|  | **O1s** _Ov_ | 531.6 | 1.6 | 26.9 |
|  | **O1s** _-OH_ | 533.0 | 1.8 | 21.4 |
|  | **O1s** _H2O_ | 534.3 | 1.9 | 4.1 |
| **MZFO_SO_2_** | **O1s** _O-Fe, O-Mn, O-Zn_ | 530.0 | 1.3 | 46.1 |
|  | **O1s** _Ov_ | 531.5 | 1.8 | 33.9 |
|  | **O1s** _-OH_ | 533.0 | 1.9 | 20.0 |

**Table S5.** XPS S 2p assignment of MZFO after gas adsorption.

| **Samples** | **Assignment** | **E_B_ (eV)** | **FWHM (eV)** | **At. %** |
| --- | --- | --- | --- | --- |
| **MZFO_ H_2_S** | **S 2p_3/2_** _S2-_ | 161.3 | 1.5 | 36.1 |
|  | **S 2p_3/2_** _S/Sn_^n-^ | 163.6 | 1.7 | 25.1 |
|  | **S 2p_3/2_** _Sulphate_ | 167.9 | 2.2 | 38.8 |
| **MZFO_SO_2_** | **S 2p_3/2_** _Sulphate_ | 168.4 | 2.2 | 100 |
